# Supplementary material for: Photo-downregulation of SIRT4 mitigates aging in mice by enhancing H3K9ac via fatty acid metabolism
Source: eLife. 2026 Oct 8;15:RP111498. doi: 10.7554/eLife.111498 (PMC13649259; doi:10.7554/eLife.111498)
Supplement: Supplementary file 2 [file elife-111498-supp1.docx]

Table A: qPCR primers used in this work.

| **Gene Names** | **Forward primer** | **Reverse primer** |
| --- | --- | --- |
| ***IL-10*** | ATGCTGCCTGCTCTTACTGACTG | CCCAAGTAACCCTTA AAGTCCTGC |
| ***Cxcl3*** | CCAGACAGAAGTCATAGCCAC | CTTCATCATGGTGAGGGGCTT |
| ***IL-1α*** | CGCTTGAGTCGGCAAAGAAAT | TGGCAGAACTGTAGTCTTCGT |
| ***IL-1b*** | GCCACCTTTTGACAGTGATGAG | GACAGCCCAGGTCAAAGGTT |
| ***IL-6*** | GACAAAGCCAGAGTCCTTCAGA | GTGACTCCAGCTTATCTCTTGG |
| ***IL-18*** | ACAAGCATCCAGGCACAGC | AAGGTTTGAGGCGGCTTTCT |
| ***Ccl2*** | AGATGCAGTTAACGCCCCAC | GAGCTTGGTGACAAAAACTACAGC |
| ***IL-20*** | TCGGGATAGTGTGTCTTTGGA | GCGAGGCTGCTGATCTTTCT |
| ***TNF-α*** | CCCTCACACTCACAAACCAC | ACAAGGTACAACCCATCGGC |
| ***Cxcl1*** | CCCAAACCGAAGTCATAGCCA | CCGTTACTTGGGGACACCTT |
| ***Cxcl2*** | CCAAAAGATACTGAACAAAGGCAAG | ATCAGGTACGATCCAGGCTTC |
| ***IL-8*** | GGCCCAATTACTAACAGGTTCC | TCTCTTGTTCTCAGGTCTCCCA |
| ***Ccl22*** | GAGATCTGTGCCGATCCCAG | CCACGGTCATCAGAGTAGGC |
| ***Ccl17*** | AATGTAGGCCGAGAGTGCTG | GACAGTCAGAAACACGATGGC |

Table B: ChIP-qPCR primers used in this work.

| Gene Names | | Forward primer | Reverse primer |
| --- | --- | --- | --- |
| **SOD2** | -208/-353 | ggtgaaggtgcagccatagt | aagggctggcatcacttctc |
|  | -1745/-1861 | aggggccctgattactccat | gtgagctgcaaagcttccac |
|  | -1078/-1238 | ctgccatgctcccaccttaa | tcagaccggagctgtatgga |
| **Cdkn2a(P21)** | -1908/-2107 | gtctgttcagtcctgggtgg | ggcaaagtgggacgtcctta |
|  | -256/-449 | ggctcacttacagttccccc | gacactctgctccacacaca |
|  | -1684/-1892 | tcccctgtccttttctggga | tccgattttgctgctggtct |
| **Cdkn2a(P16)** | -1933/-2072 | caggtcaggagcagagtgtg | gatggctctcctcgagttcg |
|  | -1290/-1480 | ttgccctccgatgacttcac | agctacaacttcgccaagct |
|  | -1022/-1238 | aggcagaagggagacagagt | ttggtgtgctgcgcataaac |
| **PGC-1α** | -1502/-1783 | ggggtgttgccttcaaacac | gccaccaactctaaaccgga |
|  | -1764/-1961 | tccggtttagagttggtggc | cctcccttctcctgtgcaag |
|  | -719/-1001 | ggtcccctgtgcatttctca | ctccaaccctagtgccttgg |
| **LAMB1** | -1346/-1501 | aacggaaaacagagggtgca | cccacttttggctgtgtgtg |
|  | -1003/-1144 | ggaaccactccacgtgtagg | ggtttttgcttcgtcacccc |
|  | -1840/-2074 | gcccgagggtcagattttga | ccgcagcactttgtttcctc |
