# Supplementary material for: Photo-downregulation of SIRT4 mitigates aging in mice by enhancing H3K9ac via fatty acid metabolism
Source: eLife. 2026 Oct 8;15:RP111498. doi: 10.7554/eLife.111498 (PMC13649259; doi:10.7554/eLife.111498)
Supplement: Supplementary file 3 [file elife-111498-fig1-data1.zip › Figure 1 - Source data 1/Figure 1 - Source data 1.pdf]

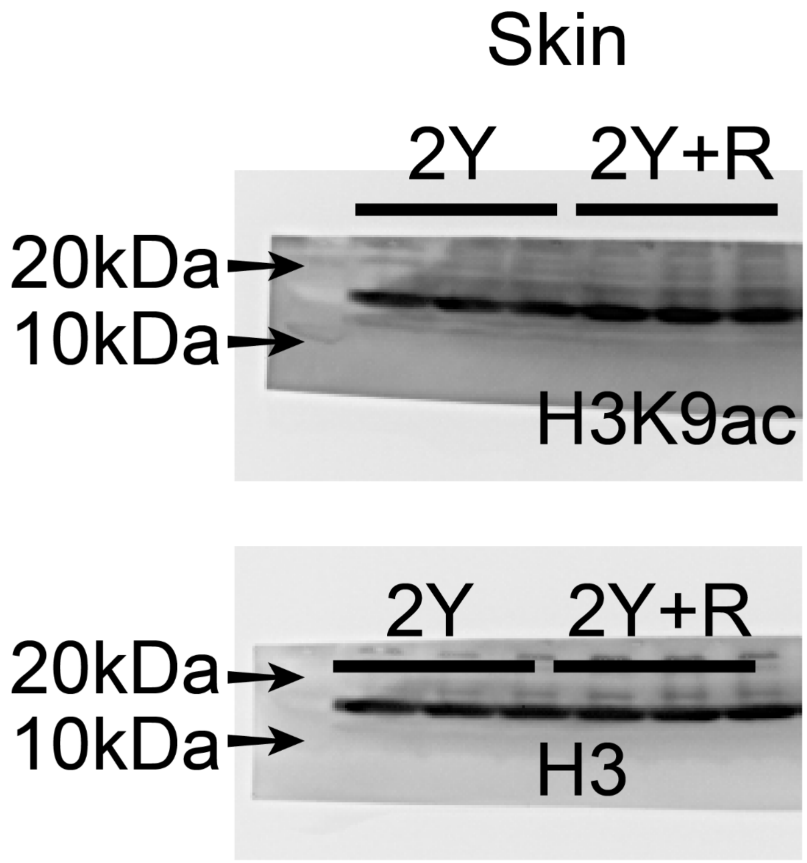

Figure 1, Source Data 1. Original membranes corresponding to Figure 1. Lanes 1, 2 and 3 correspond to skin from two-year-old C57 mice, whilst lanes 4, 5 and 6 correspond to skin from two-year-old C57 mice treated with periodic red light irradiation.
