# Supplementary material for: Photo-downregulation of SIRT4 mitigates aging in mice by enhancing H3K9ac via fatty acid metabolism
Source: eLife. 2026 Oct 8;15:RP111498. doi: 10.7554/eLife.111498 (PMC13649259; doi:10.7554/eLife.111498)
Supplement: Supplementary file 5 [file elife-111498-fig1-figsupp1-data1.zip › Figure 1 - figure supplement 1 - Source data 1/Figure 1 - figure supplement 1 - source data 1.pdf]

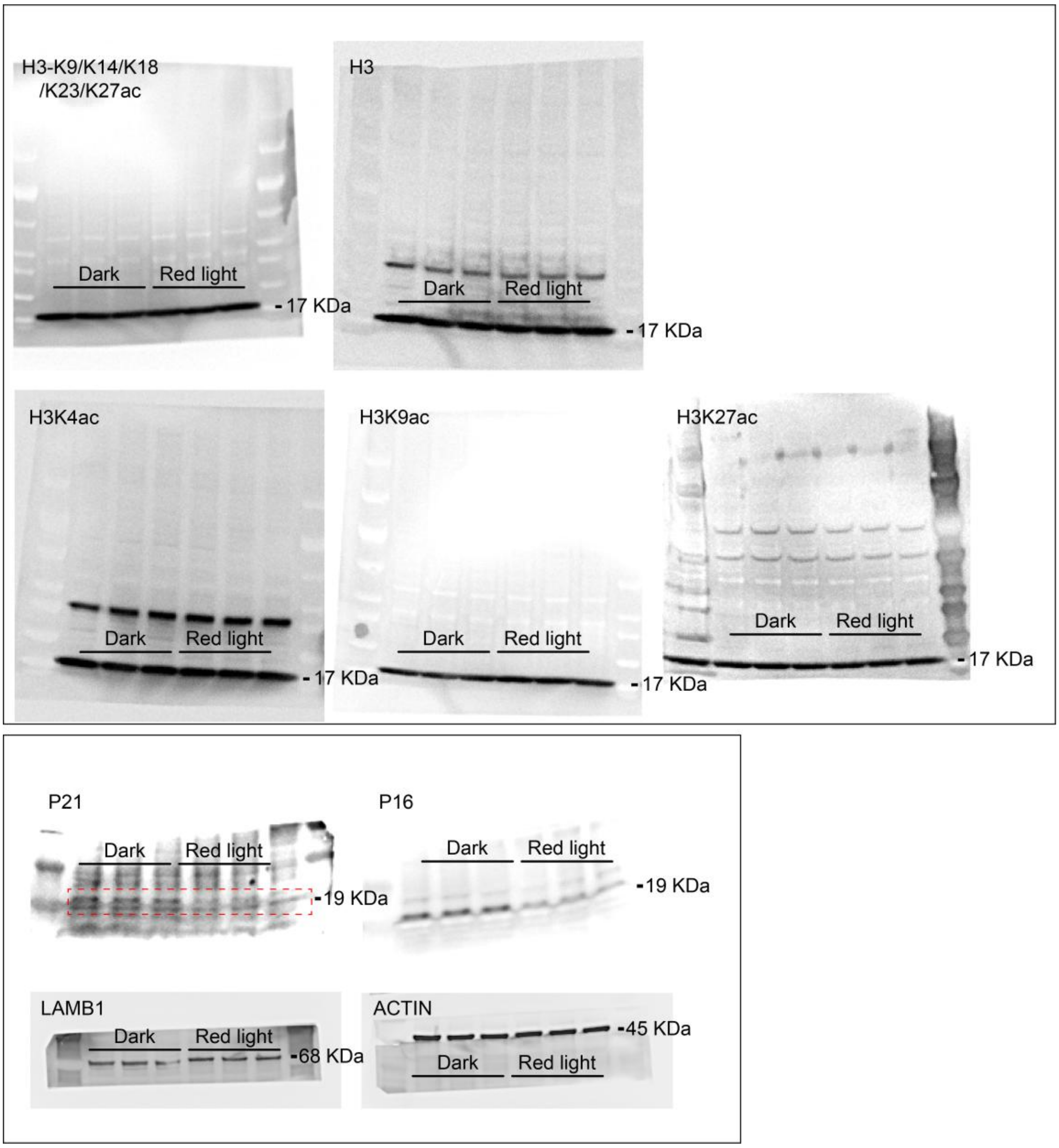

Figure 1, figure supplement 1, Source Data 1. Original membranes corresponding to figure supplement 1. Lanes 1, 2 and 3 correspond to keratinocytes from the control group, whilst lanes 4, 5 and 6 correspond to keratinocytes treated with 80 J/cm² of red light irradiation.
