# Supplementary material for: Photo-downregulation of SIRT4 mitigates aging in mice by enhancing H3K9ac via fatty acid metabolism
Source: eLife. 2026 Oct 8;15:RP111498. doi: 10.7554/eLife.111498 (PMC13649259; doi:10.7554/eLife.111498)
Supplement: Supplementary file 11 [file elife-111498-fig4-data1.zip › Figure 4 - Source data 1/Figure 4 - Source data 1.pdf]

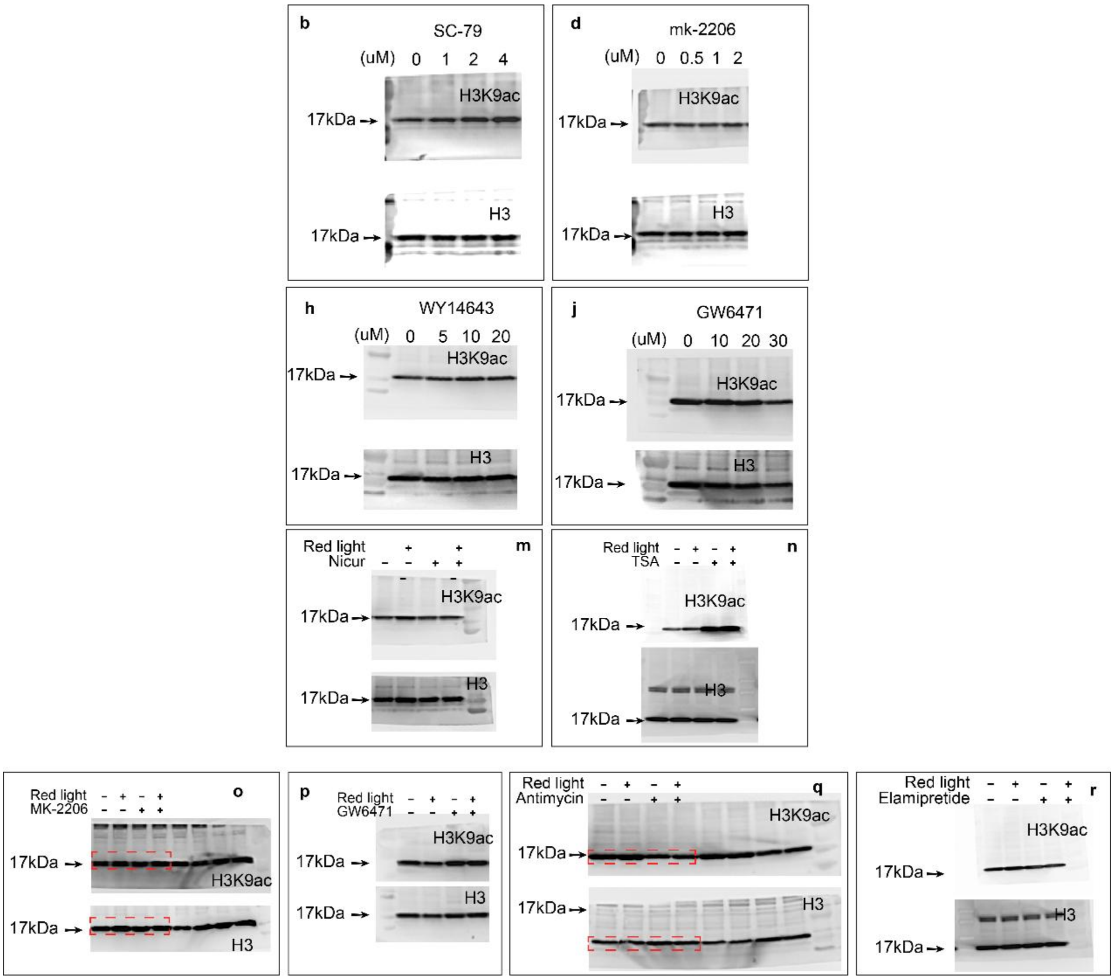

Figure 4, Source Data 1. Original membranes corresponding to Figure 4.
