# Supplementary material for: Photo-downregulation of SIRT4 mitigates aging in mice by enhancing H3K9ac via fatty acid metabolism
Source: eLife. 2026 Oct 8;15:RP111498. doi: 10.7554/eLife.111498 (PMC13649259; doi:10.7554/eLife.111498)
Supplement: Supplementary file 17 [file elife-111498-fig5-figsupp1-data1.zip › Figure 5 - figure supplement 1 - Source data 1/Figure 5 - figure supplement 1 - Source data 1.pdf]

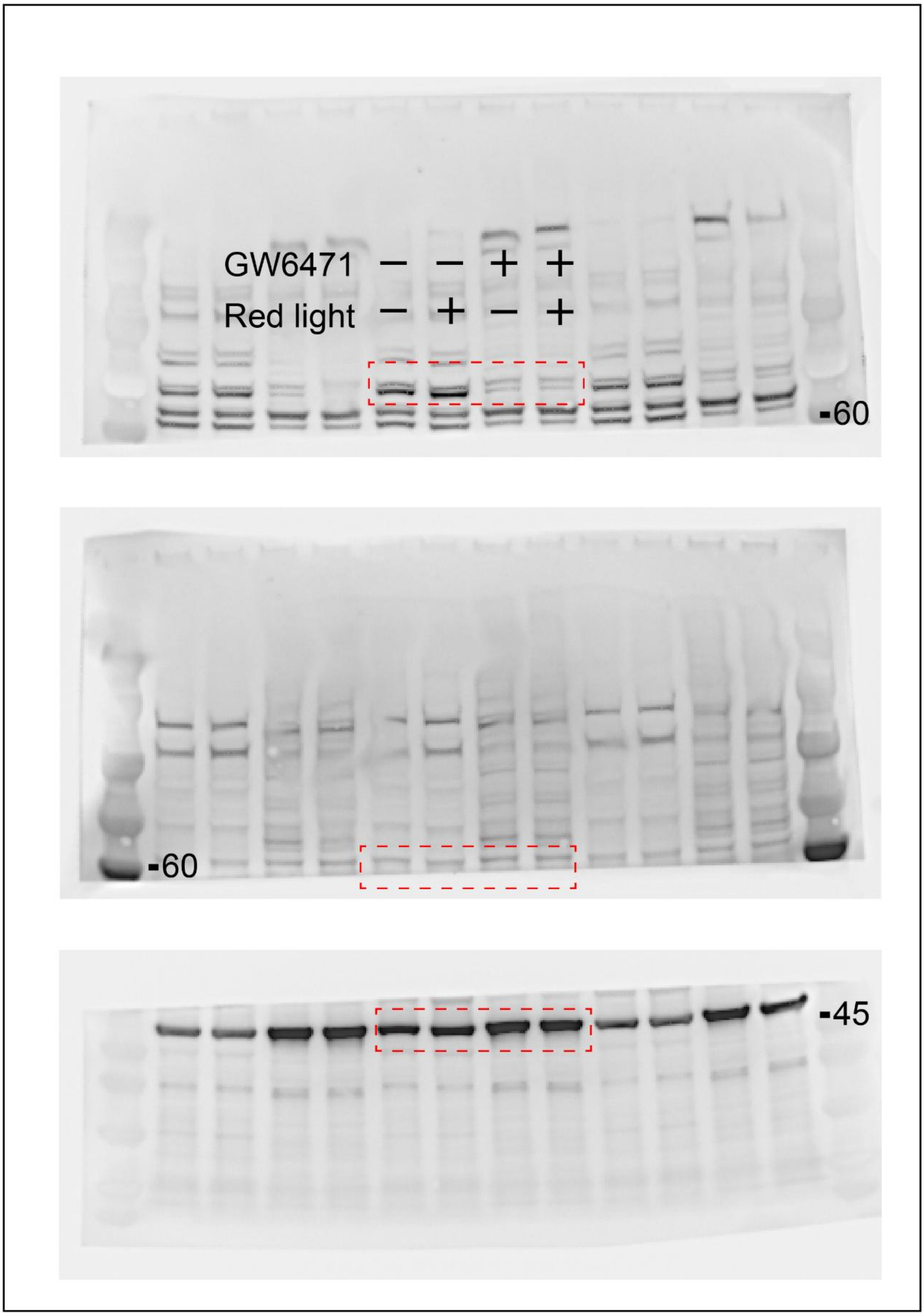

Figure 5, figure supplement 1, Source data 1, Source Data 1. Original membranes corresponding to Figure 5, figure supplement 1.
