# Supplementary material for: Photo-downregulation of SIRT4 mitigates aging in mice by enhancing H3K9ac via fatty acid metabolism
Source: eLife. 2026 Oct 8;15:RP111498. doi: 10.7554/eLife.111498 (PMC13649259; doi:10.7554/eLife.111498)
Supplement: Peer review history

# Editor report: eLife Assessment

**DOI:** 10.7554/eLife.111498.3.sa0

**Authors:** Marcelo A Mori

**Affiliations:** Universidade Estadual de Campinas (UNICAMP) Brazil

**Keywords:** Solid, Valuable

This potentially **valuable** study investigates the anti-senescence effects of red light exposure, proposing that reduced SIRT4 levels enhance fatty acid metabolism and H3K9ac, thereby attenuating ageing-related phenotypes. The authors use multiple approaches, including cultured cells, animal models, and molecular analyses, to support their conclusions. Following revision, many of the concerns previously raised by the reviewers have been addressed. The evidence is **solid**, whereas additional controls and stronger mechanistic data are still needed to fully substantiate the proposed pathway, particularly the mechanism by which red light exposure leads to reduced SIRT4 levels.

# Referee report: Reviewer #1 (Public review)

**DOI:** 10.7554/eLife.111498.3.sa1

Summary:

Deng and colleagues pursue the possibility that red light exposure can provide some benefits and anti-senescence effects in aged mouse models. In addition, they show how red light influence metabolism in cultured keratinocytes. The authors provide a long dissection of the potential paths involved in the changes promoted by red light exposure, identifying CytC oxidase, SIRT4, PPARa and MCD as key players.

Strengths:

The authors did a thorough exploration of the multiple potential avenues by which red light exposure influence metabolism. The in vitro and in vivo evidence nicely complement each other.

Weaknesses:

This is a challenging hypothesis that would require some additional experimental controls. The pathway dissection, while extensive, sometimes is approach in unconvincing ways and the results are not always evident to judge or interpret. Technically, the western blots and transcriptomic analyses require notable improvements.

Comments on revised version.

The revised version of the manuscript provides some improvements. However, I feel that many aspects remain poorly addressed. In the authors' favour, many of these limitations are now acknowledged in their rebuttal, as well as in the discussion section.

# Referee report: Reviewer #2 (Public review)

**DOI:** 10.7554/eLife.111498.3.sa2

Summary:

This work identifies a previously unknown way that red light can slow ageing. The authors show that red light lowers the level of a protein called SIRT4 in skin cells. Reducing SIRT4 boosts fatty acid use and increases a type of histone modification that keeps genes active. These changes help cells clear away signs of ageing, reduce inflammation, and restore normal metabolism. The findings open the possibility of developing new treatments that target SIRT4 to reverse age‑related decline.

Strengths:

The evidence is solid because the authors use several complementary methods. They test red light in both cultured cells and naturally aged mice, and they confirm the key role of SIRT4 by silencing its gene. Measurements of metabolism, protein changes, and ageing markers all point in the same direction. However, the exact way red light lowers SIRT4 levels is not fully explained, which leaves a minor gap. Overall, the conclusions are well supported and convincing.

Weaknesses:

The paper does not evolve to use the mechanistic discoveries of the manuscript to help our community to identify the mechanism of photobiomodulation, which is not known so far.

I would like to draw your attention to a recently published paper by Herrera et al. (FEBS Letters 2025, doi:10.1002/1873-3468.70195), which shows that red light (660 nm) stimulates mitochondrial fatty acid oxidation in keratinocytes via AMPK‑dependent phosphorylation of ACC, without altering expression of electron transport chain complexes. I believe this paper is highly complementary to current study.

Herrera et al. demonstrate that red light increases basal, ATP‑linked, and maximal oxygen consumption rates in keratinocytes specifically through enhanced fatty acid oxidation (inhibited by etomoxir). This independently validates the central finding of the current manuscript ,i.e., red light boosts lipid metabolism, strengthening the robustness of this concept.

While the current manuscript focusses on the SIRT4‑MCD axis, Herrera et al. identify AMPK phosphorylation and ACC inhibition as key effectors. Authors can integrate and expand their discussion, since SIRT4 downregulation may converge on AMPK activation, or they may represent parallel, reinforcing mechanisms. This would enrich the mechanistic model and open new hypotheses.

The mechanism of photobiomodulation: Herrera et al. explicitly challenge the prevailing paradigm that red light acts solely via cytochrome c oxidase (by showing long‑lasting effects, unchanged OXPHOS protein levels, and no difference in permeabilized cells). The current finding red light acts through SIRT4 downregulation, i.e., not direct enzymatic activation, aligns perfectly with Herrera´s critique.

Long‑term metabolic effects - Herrera et al. show that a single red light exposure elevates oxygen consumption for up to 2 days. The current study focuses on changes at 12‑24 h. Their data extend the time window and suggest that the metabolic reprogramming you describe may persist longer than currently discussed, which is clinically relevant.

Discussing Herrera et al. results would not only acknowledge independent, corroborating evidence but also allow the authors to position your SIRT4‑centric mechanism within a broader, emerging understanding of red‑light photobiomodulation.

Comments on the latest version:

The authors have made a terrific work in answering the reviewers and modifying the manuscript.

# Author comment: Author response

**DOI:** 10.7554/eLife.111498.3.sa3

**Authors:** Fangqing Deng, Rong Yang, Jinyun Niu, Monian Wang, Yang Liu, Xu Li, Zibo Gao, Lihua Yang, Huifang Liu, Yingchun Yang, Zhaoxiang Yu, Lianbing Zhang

**Affiliations:** Northwestern Polytechnical University Xi'an China; Northwestern Polytechnical University Xi'an China; Northwestern Polytechnical University Xi'an China; Northwestern Polytechnical University Xi'an China; Northwestern Polytechnical University Xi'an China; Northwestern Polytechnical University Xi'an China; Northwestern Polytechnical University Xi'an China; Northwestern Polytechnical University Xi'an China; Northwestern Polytechnical University Xi'an China; The First Affiliated Hospital of Xi'an Medical University Xi'an China; The First Affiliated Hospital of Xi'an Medical University Xi'an China; Northwestern Polytechnical University Xi'an China

The following is the authors’ response to the original reviews.

We sincerely thank the editors and reviewers for your careful evaluation of our manuscript and for the constructive recommendations that have helped us improve the rigor, clarity, and balance of the study. We are pleased that the reviewers recognized the potential value of linking red light exposure to SIRT4 downregulation, fatty acid metabolism, H3K9 acetylation, and attenuation of ageing-related phenotypes. We have revised the manuscript extensively in response to the reviewers’ comments.

In particular, we have clarified the wavelength specificity of the red-light response, reanalyzed and more cautiously interpreted the omics data, improved the presentation and quantification of semi-quantitative experiments, revised statistical reporting, corrected gene/pathway annotations, toned down mechanistic claims where direct evidence was insufficient, and expanded the Discussion to integrate recent evidence on red-light-induced fatty acid oxidation and AMPK/ACC signaling. We also added a dedicated limitations paragraph addressing the use of female mice, the absence of a complete in vivo wavelength-control and source-blocked sham cohort, and the need for future direct metabolic flux and isolated mitochondria studies.

**Public Reviews:**

**Reviewer #1 (Public review):**

Weaknesses:

This is a challenging hypothesis that would require some additional experimental controls. The pathway dissection, while extensive, is sometimes approached in unconvincing ways, and the results are not always evident to judge or interpret. Technically, the western blots and transcriptomic analyses require notable improvements.

We would like to thank the reviewer for the careful and patient examination of the issues identified in our manuscript. The poor quality of some of the Western blot bands in Figure 4 may have been caused by inappropriate electrophoresis conditions during the Western blot experiments. In the revised manuscript, we will optimize the electrophoresis conditions to obtain higher-quality protein bands and update the quantitative data. Regarding the quantification format, we believe that heatmaps provide a more intuitive representation of trends in protein expression across different treatment groups. This approach more accurately reflects the results of our biological replicates than simply analyzing the significance of differences in the grayscale values of protein bands. For the analysis of transcriptomic data, we will conduct a more detailed analysis of signal pathway enrichment and the identified differentially expressed genes to ensure that predicted genes are excluded from our current results and redundant data presentation is removed.

Regarding additional experimental controls, such as incorporating experimental data under blue light treatment conditions as a control for red light. While exploring the optimal red light irradiation dose at the cellular level, we simultaneously conducted experiments on the effects of blue light irradiation at the same dose on keratinocyte activity. The results indicated that as the blue light irradiation dose increased (0–160 J/cm^2^), the keratinocyte activity exhibited a dose-dependent decline. This indicates that blue light is phototoxic to keratinocytes. The relevant experimental results have already been published in our previous study (Communications Biology 2024, doi: 10.1038/s42003-024-06973-1). Taken together with the data from our study, this demonstrates that the anti-ageing effects of red light reported in the current manuscript are indeed driven by red light.

**Reviewer #2 (Public review):**

Weaknesses:

The paper does not evolve to use the mechanistic discoveries of the manuscript to help our community to identify the mechanism of photobiomodulation, which is not known so far.

I would like to draw attention to a recently published paper by Herrera et al. (FEBS Letters 2025, doi:10.1002/1873-3468.70195), which shows that red light (660 nm) stimulates mitochondrial fatty acid oxidation in keratinocytes via AMPK‑dependent phosphorylation of ACC, without altering expression of electron transport chain complexes. I believe this paper is highly complementary to the current study.

Herrera et al. demonstrate that red light increases basal, ATP-linked, and maximal oxygen consumption rates in keratinocytes specifically through enhanced fatty acid oxidation (inhibited by etomoxir). This independently validates the central finding of the current manuscript, i.e., red light boosts lipid metabolism, strengthening the robustness of this concept.

While the current manuscript focuses on the SIRT4-MCD axis, Herrera et al. identify AMPK phosphorylation and ACC inhibition as key effectors. The authors can integrate and expand their discussion, since SIRT4 downregulation may converge on AMPK activation, or they may represent parallel, reinforcing mechanisms. This would enrich the mechanistic model and open new hypotheses.

The mechanism of photobiomodulation: Herrera et al. explicitly challenge the prevailing paradigm that red light acts solely via cytochrome c oxidase (by showing long-lasting effects, unchanged OXPHOS protein levels, and no difference in permeabilised cells). The current finding (red light acts through SIRT4 downregulation, i.e., not direct enzymatic activation) aligns perfectly with Herrera´s critique.

Long-term metabolic effects-Herrera et al. show that a single red light exposure elevates oxygen consumption for up to 2 days. The current study focuses on changes at 12-24 h. Their data extend the time window and suggest that the metabolic reprogramming you describe may persist longer than currently discussed, which is clinically relevant.

Discussing Herrera et al.'s results would not only acknowledge independent, corroborating evidence but would also allow the authors to position their SIRT4-centric mechanism within a broader, emerging understanding of red-light photobiomodulation.

We would like to thank the reviewer for providing us with constructive suggestions for discussion. Our results showed that under red light conditions, both glycolipid and lipid metabolism were activated in keratinocytes, and cellular metabolic flux increased. The activation of lipid metabolism directly led to an increase in metabolism-associated H3K9ac and drove the upregulation of anti-ageing-related genes; we believe this is key to the anti-ageing effects of red light. Mechanistic analysis combining proteomics and acetylation proteomics revealed that red light significantly downregulated SIRT4 expression and increased the acetylation of MCD, a protein regulated by SIRT4 that governs cellular fatty acid oxidation rates. Through validation using cell-level knockdown and inhibitors, we confirmed that SIRT4 inhibition exerts anti-ageing effects in vitro and that inhibiting MCD function under red light conditions suppresses H3K9ac. These results establish the role of the SIRT4-MCD signalling axis in mediating the anti-ageing effects of red light.

The study by Herrera et al. included a substantial body of validation data confirming the role of red light in promoting fatty acid oxidation, providing robust empirical support for our research. Furthermore, Herrera et al. revealed that red light-induced fatty acid oxidation depends on AMPK and ACC phosphorylation. This mechanism of red-light photobiomodulation may refute the notion that its bio-regulatory effects rely solely on the action of mitochondrial cytochrome c oxidase. Furthermore, together with our study revealing that red light exerts anti-ageing photobiomodulatory effects via the SIRT4-MCD signalling axis, these findings independently confirm that red light regulates cellular fatty acid oxidation, thereby demonstrating the pivotal role of activated fatty acid oxidation in the bio-regulatory effects of red light. In the revised manuscript, we will include a discussion on the potential link between the red light-driven downregulation of SIRT4 and the phosphorylation of AMPK/ACC. This will be of positive value in elucidating how SIRT4 exerts its anti-ageing effects by regulating lipid metabolism, as well as in explaining the possible mechanisms by which red light downregulates SIRT4.

**Recommendations for the authors:**

Summary of Major Revisions

Changes made in the revised manuscript:

(1) Added a clearer explanation of why the 625-635 nm red-light regimen was considered the active intervention and how the available blue-light data from our previous work support wavelength-dependent effects on keratinocytes.

(2) Revised the language describing inflammatory regulation. We now avoid presenting red light as producing a uniform anti-inflammatory effect and instead describe selective remodeling of ageing-associated inflammatory and SASP signatures.

(3) Improved figure presentation and quantification for immunofluorescence, metabolite, and western blot assays; clarified image-analysis regions, replicate numbers, and normalization procedures.

(4) Reanalyzed transcriptomic, proteomic, and acetyl-proteomic datasets with appropriate multiple-testing correction and corrected erroneous pathway/gene annotations in metabolic gene panels.

(5) Replaced overly strong causal wording with more conservative language, especially regarding PI3K/Akt/mTOR, cytochrome c oxidase, SIRT4 localization, PPARα immunofluorescence, and direct fatty acid oxidation flux.

(6) Expanded the Discussion to incorporate Herrera et al. (FEBS Letters 2025, doi:10.1002/1873-3468.70195), highlighting convergence between the SIRT4-MCD model and AMPK/ACC-dependent fatty acid oxidation.

(7) Corrected typographical, nomenclature, and figure-legend inconsistencies throughout the manuscript.

**Reviewer #1 (Recommendations for the authors):**

(1) Wavelength specificity and need for a non-red-light control

As a reader, one is left wondering whether the effects are due to red light specifically. An important control would have been to irradiate mice and cells with another light wavelength, such as blue light.

We agree that wavelength specificity is a critical issue for interpreting photobiomodulation studies. In the revised manuscript, we have clarified that the anti-ageing and metabolic effects described here apply specifically to our 625-635 nm red-light regimen, rather than to visible light in general. We have also added a discussion of our previously published blue-light experiments, in which keratinocyte viability decreased in a dose-dependent manner across the same 0-160 J/cm^2^ dose range (Communications Biology 2024, doi: 10.1038/s42003-024-06973-1). These data indicate that blue light and red light produce distinct biological outcomes in keratinocytes. Because high-dose blue light was cytotoxic under comparable cellular conditions and because the present study was designed to investigate the long-term effects of red light in aged mice, we did not perform prolonged in vivo blue-light irradiation as an ageing intervention.

Changes made in the revised manuscript:

Clarified in the revised Introduction and Discussion that the conclusions are specific to 625-635 nm red light under the irradiation parameters used in this study. (Lines 92 to 94, Lines 1146-1149)

Added text summarizing the published blue-light comparison data from our previous study (Communications Biology 2024, doi: 10.1038/s42003-024-06973-1), including the dose-dependent decline in keratinocyte activity after blue-light irradiation. (Lines 90 to 92, Lines 1146-1149)

Added data on the wavelength range of the red light used in this study. (Lines 529 to 531, Fig S1a)

(2) Complexity of inflammatory effects

The manuscript repeatedly emphasizes anti-inflammatory effects, yet some cytokines such as IL-18, Ccl2, TNF-α, Ccl2, and IL-8 appear increased. This suggests that the effects may be more complex than presented and may require additional readouts or stronger statistical power.

We unanimously agree that the inflammatory response to red light should not be described as a simple, uniform suppression of all cytokines. We have demonstrated that changes in the levels of the senescence-associated secretory phenotype (SASP) at the cellular level and in skin tissue following red light treatment not only indicate that red light-induced metabolic activation can reduce the age-related inflammatory baseline, but also reveal red light-driven short-term reparative effects or stress-related cytokine responses. We consider this to be consistent with the findings, and the downregulation of NF-κB-related signalling observed in skin tissue following periodic red light irradiation of aged mice further supports the conclusion that red light alleviates the age-related inflammatory baseline. In fact, in our previous study, we did observe that red light treatment promoted increased levels of the cytokine Ccl2, which plays an important positive role in rapid wound healing (Communications Biology 2024, doi: 10.1038/s42003-024-06973-1). In the revised manuscript, we have reworded the relevant Results and Discussion sections to indicate that red light remodels ageing-associated inflammatory signalling rather than globally reducing every inflammatory mediator. We have also toned down statements suggesting that red light ‘reverses’ or ‘suppresses’ inflammation where the underlying data support a more selective effect.

Changes made in the revised manuscript:

Replaced broad terms such as “anti-inflammatory effects” with more precise wording such as “remodeling of ageing-associated inflammatory signaling” where appropriate. (Lines 541 to 543, Lines 573 to 574, Lines 893 to 895)

Expanded the Discussion to explain that red-light-induced metabolic activation may simultaneously reduce senescence-associated inflammatory tone while allowing transient reparative or stress-related cytokine responses. (Lines 1135 to 1142)

(3) Figure clarity, semi-quantitative methods, western blot quality, and inconsistent band patterns

Many differences are difficult to see or require orthogonal validation. Some tissue-specific signals and western blots are difficult to judge. Several western blots are of poor quality, and multiple markers show inconsistent band profiles across experiments, including SIRT4 in Figure 5.

We thank the reviewer for highlighting these technical and presentation issues. We have reviewed the semi-quantitative data and revised the presentation of the figures to improve their interpretability. For Western blot experiments, we optimised the electrophoresis and transfer conditions, replaced low-quality representative images where possible, and updated the semi-quantitative results. Furthermore, regarding the lack of clarity in the SIRT4 protein band, we have conducted repeat experiments and updated the main text to include a clearer image of the band. The issue with the annotation of the protein location was in fact due to an oversight during the data analysis process; we have carried out a detailed review and provided the uncropped full-length Western blot images for all experiments in the Supplementary Materials for the reviewers’ scrutiny. Finally, we would also like to point out that factors such as sample origin, protein extraction, electrophoresis conditions, antibody exposure time, and potential non-specific detection may all contribute to differences in band patterns. At present, the core conclusions regarding SIRT4 are supported by multiple lines of evidence, including mRNA analysis, immunofluorescence, Western blotting of bands at the expected sizes, and SIRT4 knockdown experiments, rather than being based solely on any single semi-quantitative Western blot result. We therefore believe that the conclusions drawn from the data presented in the revised manuscript are equally convincing.

Changes made in the revised manuscript:

Replaced or improved low-quality Western blot panels and updated quantitative analyses in revised Figures 3-5 and associated supplementary material. (Fig 3m, Fig 4, Fig 5f)

Clarified the normalization approach for H3K9ac/H3 and target/loading-control comparisons, and the use of Actin or H3 as appropriate loading controls. (Lines 283 to 293)

Bands with nonspecific profiles were excluded from quantitative conclusions and the manuscript conclusions no longer depend on those ambiguous signals.

Revised the Results (Repeat the experiment to update the low-quality Bands) to avoid overstating changes that are not clearly visible or not supported by statistical analysis. (Fig 4n and r)

(4) Choice of pharmacological agents and need for genetic strategies

The choice of drugs in Figure 4 is puzzling. More specific and widely used inhibitors could be used to block PI3K/Akt or mTOR, and natural agonists such as insulin or EGF could be used. Genetic strategies should complement these observations.

We agree that pharmacological perturbation experiments should be interpreted with caution. In this study, our criteria for selecting inhibitors were based on transcriptomic and proteomic analyses; we sought to determine how the most direct inhibition of red light-activated signalling pathways would affect H3K9ac levels. In the revised manuscript, we have clarified the rationale for the compounds used and have reduced the causal weight assigned to these inhibitor/agonist experiments. These data are now presented as supportive evidence that red light is associated with metabolism-related signalling changes, rather than as definitive proof that PI3K/Akt/mTOR is the primary upstream mechanism. We have also emphasised the genetic SIRT4 knockdown experiments as a more direct mechanistic test for the SIRT4-centred part of the model. We acknowledge that additional experiments using more selective inhibitors, physiological agonists such as insulin or EGF, and genetic perturbation of PI3K/Akt/mTOR components would be valuable for future studies.

Changes made in the revised manuscript:

Revised the text describing pharmacological experiments to distinguish supportive pathway modulation from direct causal evidence. (Lines 787 to 789)

Added a limitation and future direction noting that genetic perturbation of PI3K/Akt/mTOR and physiological pathway activation with insulin or EGF would strengthen the model. (Lines 1190 to 1194)

(5) Serum NADH measurement

In Figure 1t, the authors measure serum NADH. NADH is poorly detectable in serum or plasma, and changes may reflect blood-cell lysis during collection rather than circulating NADH.

We appreciate this technical concern. We have revised the manuscript so that serum NADH is no longer used as a central mechanistic readout. We now treat this measurement only as an exploratory indicator of systemic redox-related changes and explicitly acknowledge that serum or plasma NADH is vulnerable to artifacts from blood-cell disruption during sampling. The mechanistic interpretation has been shifted toward cellular and tissue measurements, including intracellular NADH/NADPH/GSH, ATP, acetyl-CoA, fatty acid uptake, and H3K9ac, which are more directly relevant to keratinocyte metabolic remodeling.

Changes made in the revised manuscript:

Removed serum NADH from the main causal argument linking red light to metabolic flux and H3K9ac.

Placed greater emphasis on cell-based metabolite assays, tissue acetyl-CoA, and H3K9ac measurements as the main metabolic-epigenetic evidence. (Lines 582 to 587, Fig 1s)

(6) Direct assessment of glycolysis and fatty acid oxidation

The authors propose that red light increases glycolysis and fatty acid oxidation, but this could be assessed directly rather than through surrogate measures.

We agree. Our current data include multiple metabolic readouts, including glucose and fatty acid uptake, ATP, NADH/NADPH/GSH, triglycerides, fatty acids, pyruvate, lactate, acetyl-CoA, and MCD-dependent changes; however, these assays are not equivalent to direct flux measurements such as Seahorse extracellular flux analysis, isotope tracing, or etomoxir-sensitive respiration. We have therefore revised the wording throughout the manuscript to distinguish metabolic remodeling and fatty-acid-oxidation-related signatures from direct measurements of fatty acid oxidation flux. We also incorporated the recent independent work by Herrera et al., which directly measured oxygen consumption and demonstrated red-light-induced fatty acid oxidation in keratinocytes. This external evidence supports the biological plausibility of our SIRT4-MCD model while making clear which aspects are directly measured in our study and which are inferred.

Changes made in the revised manuscript:

Replaced overstrong language such as “red light increases fatty acid oxidation” with “red light promotes PPAR-α-related fatty acid metabolism pathway” where direct flux data were not measured in our experiments. (Lines 882 to 883)

Expanded the Discussion to integrate direct FAO evidence from Herrera et al. and to place the SIRT4-MCD axis within a broader red-light metabolic framework. (Lines 1169 to 1189)

(7) Incorrect annotation of metabolic genes in Figure 3e

Acss2, Aldh3b1 and Aldh3a1 are not glycolytic enzymes, Aldh3a3 does not appear to exist, and several enzymes classified as FAO are fatty acid synthesis enzymes. This questions the interpretation of the data.

We thank the reviewer for identifying these annotation errors. We have rechecked the gene names and pathway assignments in the transcriptomic analysis and corrected the metabolic gene panels. We have confirmed that Acss2 is an acetyl-CoA synthase involved in the metabolism of acetate to acetyl-CoA. The Aldh family genes, meanwhile, are associated with aldehyde metabolism and detoxification. We have made the corresponding adjustments in the manuscript. The incorrectly listed Aldh3a3 entry has been removed. Furthermore, we have categorised genes involved in fatty acid metabolism as ‘fatty acid metabolism-related genes’, rather than grouping them all under the FAO category. These revisions have significantly improved the accuracy of the metabolic interpretation.

Changes made in the revised manuscript:

Reannotated Figure 3e and the corresponding Results text to correct glycolysis, TCA cycle, pentose phosphate pathway, and fatty acid metabolism categories. (Fig 3d)

Removed the erroneous Acss2 and Aldh family genes. (Fig 3d)

Revised the metabolic model to avoid using incorrectly grouped genes as evidence for direct fatty acid oxidation. (Lines 707 to 709)

(8) Transcriptomic analysis and implausible volcano-plot p-values

The transcriptomic analysis raises concerns. For example, the volcano plot in Figure 3d appears incorrect, with -log_10_(P-value) around 300 despite n=3 biological replicates.

We thank the reviewer for pointing out this important issue. We have reopened the transcriptomic data and found that the extremely high -log_10_(P-value) in the original volcano plot were caused by the automatic replacement of very small P-values—generated during the differential expression analysis—with zero in the tabular data. To avoid misleading visualisations, we have regenerated the volcano plot using Q-values in place of the original P-values. Differentially expressed genes were defined as those with a Q-value < 0.05 and |log_2_ fold change| > 1. Furthermore, for visualisation purposes only, the upper limit for q-values was set to 1 × 10^-50^ for values below 1 × 10^-50^. This adjustment does not affect the statistical classification of differentially expressed genes but prevents over-interpretation of extremely small values. The revised volcano plots and legends have been updated accordingly. To avoid any potential misinterpretation arising from these updates, the updated volcano plots are presented in the supplementary materials.

Changes made in the revised manuscript:

Reanalyzed transcriptomic data using appropriate multiple-testing correction and revised the volcano plot. (Fig S3a)

Corrected the y-axis transformation and removed implausible -log_10_(P-value) presentation. (Fig S3a)

Updated Methods to specify the statistical workflow for transcriptomic differential expression and pathway enrichment. (Supplementary materials Lines 46 to 52)

Moved the analysis of metabolic pathways based on transcriptomic data to the supplementary material, thereby reducing the reliance of the conclusions on transcriptomic data (Fig S3b).

(9) Need to tone down mechanistic claims regarding PI3K/Akt/mTOR, cytochrome c oxidase, SIRT4, and PPARα

The mechanisms proposed must be toned down. PI3K/Akt/mTOR should not be called glycolytic pathways, the link to red light or cytochrome c oxidase is vague, SIRT4 reduction requires mitochondrial counterstaining, and PPARα appears cytosolic after SIRT4 knockdown.

We agree and have substantially revised the mechanistic language. PI3K/Akt/mTOR is no longer referred to as a ‘glycolytic pathway’; instead, it is described as a metabolism-related signalling axis that may influence glucose uptake, growth and nutrient-responsive metabolism. We have also toned down statements attributing red-light effects directly to cytochrome c oxidase, as our study primarily examines downstream metabolic and epigenetic remodelling rather than direct photoreceptor activation. With regard to SIRT4, we have revised the text to avoid interpreting changes in SIRT4 immunofluorescence alone as evidence of altered mitochondrial abundance or mitochondrial localisation. The conclusion is now based on a combination of SIRT4 mRNA levels, western blot bands of the expected size, immunofluorescence trends, and SIRT4 knockdown phenotypes. With regard to PPARα, we have re-examined the PPARα antibody used for the cellular immunofluorescence experiments. In the original Figure 5p, we mistakenly used a PPARα antibody (PPARα, Abclonal, A25296) that is only suitable for Western blot (WB) experiments; we believe this was the cause of the mislocalisation of the fluorescent signal; Consequently, we conducted new experiments using a PPARα antibody (PPARα, Abclonal, A22887) specifically designed for cellular immunofluorescence. The relevant experimental data have been corrected in the manuscript.

Changes made in the revised manuscript:

Replaced “PI3K/Akt/mTOR glycolytic pathway” with “The PI3K-AKT signalling pathway is involved in the regulation of glucose metabolism” throughout the revised manuscript. (Lines 701 to 705, Lines 809 to 810, Lines 813, Lines 1193)

Reduced mechanistic certainty around cytochrome c oxidase and framed it as a possible upstream photoreceptor rather than an experimentally proven mechanism in this study. (Lines 827 to 830, Lines 842 to 848)

Repeat the PPARα immunofluorescence staining experiment. (Fig 5p)

(10) Need for isolated mitochondria experiments and red/blue light comparison of mitochondrial respiration

If the effect of red light relies on mitochondrial cytochromes, additional proof would be needed, potentially using isolated mitochondria and comparing how red and blue light influence respiration capacity.

We agree that isolated mitochondria experiments would be an important way to test direct mitochondrial photoreception. Because the present study was designed around cellular and in vivo metabolic-epigenetic remodeling, we did not perform isolated mitochondria irradiation experiments. To address this concern, we have toned down statements implying direct cytochrome activation and revised the Discussion to distinguish between direct mitochondrial photoreceptor models and downstream metabolic reprogramming. We also added a future direction proposing isolated mitochondria or permeabilized-cell experiments comparing red and blue light effects on respiration, ATP-linked OCR, maximal respiration, and FAO-dependent respiration. The revised manuscript now emphasizes that our data support a downstream SIRT4-MCD-H3K9ac mechanism after red-light exposure, while the proximal photophysical event remains to be fully defined.

Changes made in the revised manuscript:

Added discussion of the need for isolated mitochondria, permeabilized-cell, and wavelength-comparison respiration experiments. (Lines 1194 to 1197)

**Reviewer #2 (Recommendations for the authors):**

(1) Statistical reporting, post-hoc tests, normality/equal-variance tests, exact p-values, and FDR control

The manuscript states that one-way ANOVA followed by Tukey or Dunnett tests was used, but it does not consistently specify the post-hoc correction for each figure. Normality and equal-variance tests are not reported, p-values are shown only as asterisks, and FDR control is not mentioned for transcriptomics and proteomics.

We agree that the statistical reporting needed to be more complete. We have revised the Statistics and reproducibility section and the figure legends to specify the statistical test used for each experiment, the post-hoc correction applied after ANOVA, the number of independent biological replicates, and the definition of error bars. Where multiple comparisons were performed, we now state whether Tukey’s or Dunnett’s correction was used. Regarding *P*-value presentation, we have retained the use of asterisks in the figures as visual indicators of statistical significance to maintain figure readability. For transcriptomic, proteomic, and acetyl-proteomic analyses, we have revised the Methods section to state that multiple-testing correction was performed using the Benjamini–Hochberg false-discovery-rate procedure. Adjusted *P* values or *Q* values were used for differential-expression and pathway-enrichment analyses. These revisions clarify the statistical workflow and strengthen the reproducibility of the study.

Changes made in the revised manuscript:

Revised the Statistics and reproducibility section to define statistical tests, post-hoc corrections, assumption checks, and multiple-testing correction. (Lines 509 to 522)

Updated relevant figure legends to include n values, statistical tests, post-hoc corrections, and definitions of significance symbols. (Lines 515 to 516)

Added FDR control details for RNA-seq, proteomics, acetyl-proteomics, and pathway-enrichment analyses. (Lines 450 to 455)

(2) Figure clarity and quantitative analysis of fluorescence, JC-1, metabolite, and western blot data

Several figures lack clarity or appropriate quantification. Figure 1i-j H3K9ac quantification should be based on whole-image or multiple fields; Figure 2e JC-1 should include red/green ratio quantification; Figure 2k-p metabolite data should include absolute concentrations; Figure 3j needs appropriate loading controls.

We appreciate these specific suggestions and have revised the figure presentation accordingly. For H3K9ac immunofluorescence in skin sections, we have clarified the anatomical region quantified and performed a more objective quantification using multiple fields/regions per section rather than relying on a visually selected dashed area. The dashed regions in the representative images were made clearer and the quantification criteria were added to the Methods and legend. For JC-1 staining, the bar chart on the right-hand side of the mitochondrial membrane potential fluorescence image in Figure 2e shows the quantitative data for the red/green fluorescence ratio obtained from independent experiments; compared with providing only a representative image, these data offer a more easily interpretable quantitative measure of mitochondrial membrane potential. To avoid any potential misunderstanding, we have corrected the vertical axis. For metabolite assays, we clarified normalization to cell number or protein content and revised the data presentation to include absolute or normalized concentrations where available, rather than relying solely on fold changes with variable y-axis scaling.

Changes made in the revised manuscript:

Revised Figure 1i-j quantification using multiple fields/regions per mouse section and improved dashed-region visibility. (Lines 431 to 440, Fig 1i and j)

Corrected the vertical axis of the quantitative data for the JC-1 red/green fluorescence ratio. (Fig 2e and Fig S2a)

Updated metabolite panels and/or source data to include absolute or protein-normalized values where available, and standardized y-axis interpretation. (Fig 2k-p and Fig 4s and v, Given the diversity of intracellular fatty acid and triglyceride species, absolute quantification based solely on absorbance measurements would be technically challenging and may not accurately reflect the content of each molecular component. Therefore, we presented the changes in fatty acid and glycerol levels as percentage-normalized relative absorbance values, which allowed consistent comparison among the experimental groups.)

(3) Experimental design limitations: sex of mice and sham control

Only female C57BL/6 mice were used, although aging and metabolic responses can be sex-dependent. The thermal-control argument lacks a true sham control in which mice are placed in the same apparatus with the light blocked at the source.

We agree with these points. We have added a section on limitations stating that all aged mice used in this study were female, and that sex-dependent responses to red light, SIRT4 regulation, metabolism and skin ageing should be investigated in future studies using both male and female cohorts. Furthermore, regarding the design of the non-irradiated control group: although the control mice underwent the same depilation and routine procedures, they did not receive red light irradiation. However, we also acknowledge that establishing a sham-irradiated control group with light shielding would allow for stricter control of factors such as restraint, contact with equipment and procedural stress. However, given that this experiment involved a continuous cyclic photoperiodic treatment lasting two years, we were unable to supplement the study with a control experiment involving only red light shielding. Nevertheless, based on the fact that we observed only minimal changes in the mice’s skin temperature following red light irradiation, we believe that the primary factor driving the alleviation of the skin ageing phenotype in the mice remains red light-induced.

Changes made in the revised manuscript:

Added a limitation noting that the study used female C57BL/6 mice only and that sex as a biological variable should be addressed in future studies. (Lines 1198 to 1201)

(4) Textual errors, nomenclature inconsistencies, and ChIP-qPCR normalization

Several textual errors and inconsistencies should be corrected, including Pparg1a/Ppargc1a, Ricotr/Rictor, Pi3k/PI3K, Sirt4/SIRT4 protein nomenclature, and the use of RPL30 normalization in ChIP-qPCR without showing that RPL30 is unchanged.

We thank the reviewer for their careful reading. We have corrected the typographical errors and standardised gene and protein nomenclature throughout the manuscript and figure legends. Specifically, *Ppargc1α* has been corrected to *Ppargc1a*, Ricotr to Rictor, and the capitalisation of PI3K has been standardised. We now use Sirt4 for the mouse gene and SIRT4 for the protein, applying the same convention to other genes and proteins. For ChIP-qPCR, we have revised the Methods and Results sections to describe normalisation against input and IgG controls more clearly, and to specify the role of the RPL30 locus as an internal control. We have also included data in the Supplementary Materials showing relative enrichment of H3K9ac in the RPL30 promoter region in PAM212 cells before and after red light irradiation; the results indicate that H3K9ac enrichment at the RPL30 locus remained stable across treatment groups after normalization to input DNA and correction against IgG background. This result indicates that the use of RPL30 as an internal control in ChIP-qPCR experiments is feasible.

Changes made in the revised manuscript:

Corrected Ppargc1a, Rictor, PI3K, Sirt4/SIRT4, and related nomenclature throughout the manuscript.

Supplement the experimental results on the effect of red-light irradiation on the level of H3K9ac enrichment at the RPL30 locus in keratinocytes. (Lines 339-352, Lines 539 to 541, Fig S1d)

(5) Additional Revision Addressing the Public Review and Herrera et al.

The reviewer suggested integrating the recent study by Herrera et al. showing that 660 nm red light stimulates mitochondrial fatty acid oxidation in keratinocytes through AMPK-dependent phosphorylation of ACC, without changing electron transport chain complex expression. The reviewer also noted that these findings may complement the SIRT4-MCD axis and challenge a cytochrome-c-oxidase-only model of photobiomodulation.

We are grateful for this constructive suggestion. We have expanded the Discussion to incorporate Herrera et al. and to place our SIRT4-MCD-centered mechanism within the broader emerging model of red-light-driven metabolic remodeling. Herrera et al. provide direct oxygen-consumption evidence that red light enhances fatty acid oxidation in keratinocytes and that this effect involves AMPK/ACC signaling. This is highly complementary to our data, in which red light decreases SIRT4, increases acetylation of MCD, promotes fatty-acid-metabolism-related signatures, elevates acetyl-CoA, and increases H3K9ac. In the revised Discussion, we propose two nonexclusive models: red light-induced SIRT4 downregulation may converge with AMPK/ACC-dependent relief of fatty acid oxidation, or the two pathways may represent parallel reinforcing mechanisms that together enhance lipid metabolic flux.

Changes made in the revised manuscript:

Added a paragraph discussing Herrera et al. in the revised Discussion. (Lines A1169 to 1189, Lines 1206 and 1208)

Revised the conceptual model of red-light photobiomodulation to emphasize downstream metabolic reprogramming rather than direct cytochrome c oxidase activation alone. (Lines 827 to 830, Lines 843 to 844)

Added future directions to test whether red-light-induced SIRT4 downregulation causally affects AMPK/ACC phosphorylation and FAO-dependent respiration. (Lines 1169 to 1189)

We again thank the editors and reviewers for their thoughtful and constructive comments. The revised manuscript now provides a more rigorous and balanced presentation of the evidence, distinguishes direct measurements from inferred metabolic flux, corrects pathway annotations, improves figure quantification and statistical transparency, and places the SIRT4-MCD-H3K9ac mechanism within a broader framework of red-light-induced fatty acid metabolic remodeling. We believe these revisions substantially strengthen the manuscript and clarify both the significance and the limitations of our findings.
